# Supplementary material for: Targets of complement-fixing antibodies in protective immunity against malaria in children
Source: Nat Commun. 2019 Feb 5;10:610. doi: 10.1038/s41467-019-08528-z (PMC6363798; doi:10.1038/s41467-019-08528-z)
Supplement: Supplementary file 1 — Supplementary Information [file 41467_2019_8528_MOESM1_ESM.pdf]

Supplementary Data

Targets of complement-fixing antibodies in protective immunity against malaria in children

Linda Reiling[1], Michelle J. Boyle[1], Michael White[2], Danny W. Wilson[3,1], Gaoqian Feng[1,4], Rupert Weaver[1], D. Herbert Opi[1,7], Kristina E. M. Persson[5], Jack S Richards[1,4], Peter M. Siba[6], Freya J. I. Fowkes[1,4,7], Eizo Takashima[8], Takafumi Tsuboi[8], Ivo Mueller[2,6,9], James G. Beeson[1,4,7]

[1] Burnet Institute, Melbourne, Victoria, Australia
[2] Institute Pasteur, Paris, France
[3] Research Centre for Infectious Diseases, School of Biological Sciences, University of Adelaide, Adelaide, Australia
[4] University of Melbourne, Department of Medicine (Royal Melbourne Hospital) and Melbourne School of Population and Global Health, Victoria, Australia;
[5] Department of Laboratory Medicine, Lund University, Skåne University Hospital, 22185, Lund, Sweden;
[6] Papua New Guinea Institute of Medical Research, Goroka, Papua New Guinea
[7] Monash University, Central Clinical School (Infectious Diseases; Immunology; Epidemiology and Preventative Medicine) and Department of Microbiology, Victoria, Australia;
[8] Division of Malaria Research, Proteo-Science Center, Ehime University, Matsuyama, Japan;
[9] Walter and Eliza Hall Institute, Parkville, Australia

Supplementary Table 1:
Prevalence of complement-fixing antibodies against merozoite antigens

Antigen	All	Age			Enrolment *P. falciparum* parasitemic status		
	n[1]	< 9yrs n[1]	>= 9yrs n[1]	p[3]	PCR- n[1]	PCR+ n[1]	P[2]
**Merozoites**	199	89	110	0.3	64	135	0.15
	99.5%	98.9%	100%		98.5%	100%	
**MSP1-19**	197	89	108	0.7	64	133	0.98
	98.5%	98.9%	98.2%		98.5%	98.5%	
**MSP1-42**	183	79	104	0.05	51	132	<0.0001
	92%	88%	95%		80%	98%	
**MSP2 (3D7)**	197	88	109	0.4	64	133	0.98
	98.5%	97.8%	99.1%		98.5%	98.5%	
**MSP2 (FC27)**	167	73	94	0.4	45	122	<0.0001
	84.3%	82%	86.2%		70.3%	91%	
**MSP3**	63	31	32	0.4	10	53	0.001
	31.8%	34.8%	29.4%		15.6%	39.6%	
**MSP4**	192	84	108	0.18	59	133	0.024
	95.5%	93.3%	97.3%		90.8%	97.8%	
**MSP6**	189	84	105	0.7	60	129	0.28
	95.9%	96.6%	95.5%		93.8%	97%	
**MSP7**	189	81	108	0.03	59	130	0.03
	96.4%	93.1%	99.1%		92.2%	98.5%	
**MSP9**	177	78	99	0.6	53	124	0.008
	91.7%	92.9%	90.8%		84.1%	95.4%	
**MSP10**	123	53	70	0.3	28	95	<0.0001
	64.1%	60%	67%		45%	73.1%	
**MSPDBL1**	169	72	97	0.2	52	117	0.16
	86.2%	82.8%	89%		81.3%	88.6%	
**Ripr**	135	61	74	0.7	34	101	0.004
	66.2%	67.8%	64.9%		52.3%	72.7%	
**GAMA**	187	82	105	0.5	57	130	0.003
	95.4%	94.3%	96.3%		89.1%	98.5%	
**RALP1**	166	66	100	0.002	49	117	0.03
	84.7%	75.9%	91.7%		76.6%	88.6%	
**AMA1**	196	85	111	0.012	60	136	0.001
	97.5%	94.4%	100%		92.3%	100%	
**EBA140 RII**	185	76	109	0.001	54	131	<0.0001
	93.9%	87.4%	99.1%		84.4%	98.5%	
**EBA140 RIII-V**	184	78	106	0.06	55	129	0.003
	93.4%	89.7%	96.4%		85.9%	97%	
**EBA175RIII-V**	140	51	89	0.001	36	104	0.003
	70.4%	58%	80.2%		56.3%	77%	
**EBA175RII**	191	83	108	0.043	58	133	0.003
	95.5%	92.2%	98.2%		89.2%	98.5%	
**Rh2-2030**	194	85	109	0.2	60	134	0.003
	98%	96.6%	99.1%		93.8%	100%	
**Rh5**	113	42	71	0.005	25	88	0.001
	57.4%	46.7%	66.4%		39.7%	65.7%	
**Pf113**	108	46	62	0.1	29	79	0.05
	60.3%	54.1%	66%		50%	65.3%	

Number and percentage of seropositive individuals as defined by the upper 95% confidence interval from testing samples from malaria-non-exposed controls; data for complement-fixing antibodies against whole merozoites is included for comparison [1]. Complement fixation was measured by detecting complement factor C1q

[1] n=numbers (No) analysed (No all, No <9yrs, No ≥9yrs, No PCR-, No PCR+):
179, 85, 94, 58, 121 for Pf113
192, 88, 104, 62, 130 for MSP10

193, 84, 109, 63, 130 for MSP9
196, 87, 109, 64, 132 for MSPDBL1, MSP7, GAMA, RALP1
197, 87, 110, 64, 133 for MSP6, EBA140RII, EBA140RIII-V, Rh5
198, 88, 110, 64, 133 for MSP1-42, EBA175RIII-V
198, 89, 109, 64, 134 for MSP2 (FC27) and MSP3
200, 90, 110, 65, 135 for Merozoites, MSP1-19, EBA175RII
200, 90, 100, 65, 135 for MSP2 (3D7)
201, 90, 111, 65, 136 for MSP4, AMA1
204, 90, 114, 65, 139 for Ripr
RH2-2030: 88, 110, 64, 134; MSP9: 84, 109, 63, 130; MSP10: 88, 104, 62, 130; Pf113: 85, 94, 58, 121

[2] p indicates statistical significance as determined by chi-square test (for categorical variables)

Supplementary Table 2:
Correlation between IgG and complement fixing antibodies for different merozoite antigens

**Antigen**	**Spearman's rho**	**p-value**
MSP1-19	0.571	<0.0001
MSP1-42	0.912	<0.0001
MSP2-3D7	0.891	<0.0001
MSP2-FC27	0.882	<0.0001
MSP3	0.294	<0.0001
MSP4	0.922	<0.0001
MSP6	0.825	<0.0001
MSP7	0.820	<0.0001
MSP9	0.628	<0.0001
MSP10	0.734	<0.0001
MSPDBL1	0.768	<0.0001
AMA1	0.950	<0.0001
EBA140RII	0.887	<0.0001
EBA140RRIII-V	0.892	<0.0001
EBA175RII	0.921	<0.0001
EBA175RIII-V	0.824	<0.0001
RH2-2030	0.850	<0.0001
RH5	0.484	<0.0001
Ripr	0.464	<0.0001
GAMA	0.823	<0.0001
RALP1	0.691	<0.0001

Supplementary Table 3:
Associations between complement fixing antibodies and protection against clinical malaria

**Clinical malaria**	**no adjustment**		**age location adjusted**	
	**HR (95%CI)**	**p**	**aHR (95%CI)**	**p**
**merozoites**	0.12 [0.05-0.28]	<0.0001	0.15 [0.06-0.35]	<0.0001
**MSP1-19**	0.45 [0.24-0.85]	0.01	0.49 [0.26-0.93]	0.03
**MSP1-42**	0.52 [0.29-0.94]	0.03	0.62 [0.34-1.13]	0.1
**MSP2 (3D7)**	0.33 [0.17-0.62]	0.001	0.41 [0.21-0.79]	0.008
**MSP2 (FC27)**	0.44 [0.23-0.85]	0.02	0.61 [0.31-1.23]	0.4
**MSP3**	0.8 [0.45-1.21]	0.5	0.9 [0.5-1.62]	0.7
**MSP4**	0.40 [0.22-0.74]	0.004	0.54 [0.28-1.04]	0.06
**MSP6**	0.34 [0.18-0.65]	0.001	0.42 [0.21-0.85]	0.015
**MSP7**	0.21 [0.10-0.43]	<0.0001	0.23 [0.11-0.49]	<0.001
**MSP9**	0.47 [0.26-0.88]	0.02	0.7 [0.36-1.35]	0.3
**MSP10**	0.41 [0.22-0.78]	0.007	0.49 [0.26-0.95]	0.04
**MSP DBL**	0.27 [0.13-0.53]	<0.0001	0.33 [0.16-0.68]	0.002
**Ripr**	0.37 [0.20-0.69]	0.002	0.46 [0.24-0.90]	0.02
**GAMA**	0.23 [0.12-0.45]	<0.0001	0.27 [0.14-0.53]	<0.0001
**RALP1**	0.21 [0.10-0.41]	<0.0001	0.24 [0.12-0.49]	<0.0001
**AMA1**	0.39 [0.20-0.76]	0.005	0.43 [0.22-0.83]	0.012
**EBA175RII**	0.37 [0.20-0.70]	0.002	0.46 [0.24-0.89]	0.02
**EBA175RIII-V**	0.31 [0.17-0.58]	<0.0001	0.41 [0.21-0.78]	0.007
**EBA140 RII**	0.41 [0.22-0.77]	0.005	0.43 [0.23-0.80]	0.007
**EBA140 RIII-V**	0.18 [0.08-0.36]	<0.0001	0.20 [0.10-0.43]	<0.0001
**Rh2-2030**	0.28 [0.14-0.53]	<0.0001	0.30 [0.15-0.58]	<0.0001
**Rh5**	0.30 [0.15-0.60]	0.001	0.37 [0.18-0.76]	0.007
**Pf113**	1.07 [0.59-1.92]	0.8	1.3 [0.72-2.35]	0.4

Study participants were stratified into 3 equal groups according to low, medium or high levels of antigen-specific C1q fixing antibodies. Hazard ratios were calculated by comparing those with high versus low levels of antibodies for the risk of symptomatic malaria over 6 months of follow-up (using Cox proportional hazards model); analysis was based on first episode only. Unadjusted hazard ratios (HR), and adjusted (age-adjusted and location-adjusted) hazard ratios hazard ratios (aHR) were calculated. 95% CI: 95% confidence intervals. Statistical significance is indicated as p.

Supplementary Table 4:
Association between complement fixing antibodies and protection against high-density parasitemia

Antigen	No adjustment		Age location adjusted	
	HR (95%CI)	p	aHR (95%CI)	p
**merozoites**	0.26 [0.13-0.49]	<0.0001	0.35 [0.18-0.70]	0.003
**MSP1-19**	0.55 [0.31-0.97]	0.04	0.65 [0.36-1.16]	0.1
**MSP1-42**	0.59 [0.34-1.03]	0.06	0.71 [0.4-1.26]	0.2
**MSP2 (3D7)**	0.44 [0.24-0.78]	0.005	0.6 [0.33-1.10]	0.1
**MSP2 (FC27)**	0.67 [0.38-1.17]	0.2	0.98 [0.54-1.78]	0.4
**MSP3**	1.02 [0.61-1.71]	0.9	1.2 [0.7-2.05]	0.4
**MSP4**	0.54 [0.32-0.93]	0.03	0.76 [0.42-1.39]	0.4
**MSP6**	0.52 [0.3-0.91]	0.02	0.71 [0.39-1.31]	0.3
**MSP7**	0.4 [0.22-0.73]	0.003	0.49 [0.26-0.90]	0.02
**MSP9**	0.58 [0.32-1.02]	0.06	0.92 [0.57-1.71]	1.0
**MSP10**	0.55 [0.30-0.98]	0.04	0.7 [0.38-1.27]	0.2
**MSP DBL**	0.42 [0.24-0.73]	0.002	0.56 [0.31-1.01]	0.05
**Ripr**	0.39 [0.22-0.68]	0.001	0.47 [0.26-0.86]	0.014
**GA MA**	0.46 [0.27-0.8]	0.006	0.6 [0.34-1.06]	0.08
**RALP1**	0.4 [0.22-0.71]	0.002	0.54 [0.3-1.00]	0.05
**AMA1**	0.64 [0.37-1.11]	0.1	0.76 [0.44-1.33]	0.3
**EBA175RII**	0.54 [0.31-0.93]	0.03	0.71 [0.4-1.26]	0.2
**EBA175RIII-V**	0.5 [0.29-0.87]	0.01	0.74 [0.41-1.36]	0.3
**EBA140 RII**	0.56 [0.32-0.98]	0.04	0.61 [0.35-1.08]	0.1
**EBA140 RIII-V**	0.4 [0.23-0.69]	0.001	0.49 [0.28-0.88]	0.02
**Rh2-2030**	0.48 [0.27-0.85]	0.01	0.55 [0.31-0.98]	0.04
**Rh5**	0.42 [0.24-0.76]	0.004	0.55 [0.3-1.02]	0.06
**Pf113**	1.22 [0.69-2.14]	0.5	1.52 [0.87-2.83]	0.1

High density parasitemia was defined as >5000 parasites/ul, as determined by light microscopy
Study participants were stratified into 3 equal groups according to low, medium or high levels of antigen-specific antibodies. Hazard ratios were calculated comparing those with high versus low levels of antibodies for the risk of high-density parasitemia over 6 months of follow-up (using Cox proportional hazards model); analysis was based on first episode only. Unadjusted hazard ratios (HR), and adjusted (age-adjusted and location-adjusted) hazard ratios hazard ratios (aHR) were calculated. 95% CI: 95% confidence intervals. Statistical significance is indicated as p.

Supplementary Table 5:

Association between complement-fixing antibodies and protection against clinical malaria comparing low versus medium responders or low versus high responders

		no adjustment		age location adjusted	
		**HR (95%CI)**	**p**	**aHR (95%CI)**	**p**
**merozoites**	LvM	0.56 [0.34-0.94]	0.028	0.64 [0.38-1.09]	0.1
	**LvH**	0.12 [0.05-0.28]	<0.0001	0.15 [0.06-0.35]	<0.0001
**MSP1-19**	LvM	0.89 [0.52-1.55]	0.69	0.88 [0.51-1.53]	0.7
	**LvH**	0.45 [0.24-0.85]	0.01	0.49 [0.26-0.93]	0.03
**MSP1-42**	LvM	0.59 [0.33-1.04]	0.07	0.72 [0.4-1.3]	0.3
	**LvH**	0.52 [0.29-0.94]	0.03	0.62 [0.34-1.13]	0.1
**MSP2 (3D7)**	LvM	0.46 [0.26-0.8]	0.006	0.54 [0.31-0.97]	0.038
	**LvH**	0.33 [0.17-0.62]	0.001	0.41 [0.21-0.79]	0.008
**MSP2 (FC27)**	LvM	1.02 [0.6-1.75]	0.9	1.29 [0.73-2.26]	0.4
	**LvH**	0.44 [0.23-0.85]	0.02	0.61 [0.31-1.23]	0.4
**MSP3**	LvM	0.67 [0.37-1.21]	0.2	0.76 [0.41-1.39]	0.4
	**LvH**	0.8 [0.45-1.21]	0.5	0.9 [0.5-1.62]	0.7
**MSP4**	LvM	0.52 [0.3-0.91]	0.02	0.64 [0.36-1.13]	0.1
	**LvH**	0.4 [0.22-0.74]	0.004	0.54 [0.28-1.04]	0.06
**MSP6**	LvM	0.59 [0.34-1.03]	0.07	0.7 [0.39-1.24]	0.2
	**LvH**	0.34 [0.18-0.65]	0.001	0.42 [0.21-0.85]	0.015
**MSP7**	LvM	0.56 [0.33-0.96]	0.037	0.62 [0.36-1.09]	0.09
	**LvH**	0.21 [0.1-0.43]	<0.0001	0.23 [0.11-0.49]	<0.001
**MSP9**	LvM	0.57 [0.32-1.02]	0.06	0.7 [0.38-1.28]	0.2
	**LvH**	0.47 [0.26-0.88]	0.02	0.7 [0.36-1.35]	0.3
**MSP10**	LvM	0.57 [0.32-1.02]	0.06	0.63 [0.35-1.13]	0.1
	**LvH**	0.41 [0.22-0.78]	0.007	0.49 [0.26-0.95]	0.04
**MSPDBL1**	LvM	0.66 [0.38-1.1]	0.144	0.74[0.43-1.29]	0.29
	**LvH**	0.27 [0.13-0.53]	<0.0001	0.33 [0.16-0.68]	0.002
**Ripr**	LvM	0.57 [0.33-0.99]	0.05	0.67 [0.37-1.23]	0.2
	**LvH**	0.37 [0.2-0.69]	0.002	0.46 [0.24-0.9]	0.02
**GAMA**	LvM	0.38 [0.21-0.68]	0.001	0.46 [0.25-0.83]	0.01
	**LvH**	0.23 [0.12-0.45]	<0.0001	0.27 [0.14-0.53]	<0.0001
**RALP1**	LvM	0.44 [0.25-0.77]	0.004	0.48 [0.27-0.85]	0.01
	**LvH**	0.21 [0.1-0.41]	<0.0001	0.24 [0.12-0.49]	<0.0001
**AMA1**	LvM	0.8 [0.47-1.36]	0.4	0.93 [0.54-1.62]	0.8
	**LvH**	0.39 [0.20-0.76]	0.005	0.43 [0.22-0.83]	0.012
**EBA175RII**	LvM	0.56 [0.32-0.96]	0.04	0.61 [0.35-1.07]	0.08
	**LvH**	0.37 [0.2-0.7]	0.002	0.46 [0.24-0.89]	0.02
**EBA175RIII_**	LvM	0.44 [0.25-0.78]	0.005	0.52 [0.29-0.93]	0.03
**V**	**LvH**	0.31 [0.17-0.58]	<0.0001	0.41 [0.21-0.78]	0.007
**EBA140RII**	LvM	0.56 [0.32-0.99]	0.045	0.66 [0.36-1.21]	0.18
	**LvH**	0.41 [0.22-0.77]	0.005	0.43 [0.23-0.80]	0.007
**EBA140RIII-V**	LvM	0.46 [0.26-0.79]	0.005	0.53 [0.3-0.94]	0.03
	**LvH**	0.18 [0.08-0.36]	<0.0001	0.20 [0.1-0.43]	<0.0001
**Rh2-2030**	LvM	0.5 [0.28-0.87]	0.014	0.54 [0.3-0.95]	0.031
	**LvH**	0.28 [0.14-0.53]	<0.0001	0.3 [0.15-0.58]	<0.0001
**Rh5**	LvM	0.98 [0.58-1.66]	0.9	1.11 [0.65-1.89]	0.7
	**LvH**	0.3 [0.15-0.6]	0.001	0.37 [0.18-0.76]	0.007
**Pf113**	LvM	0.84 [0.45-1.58]	0.6	1.0 [0.53-1.88]	1
	**LvH**	1.07 [0.59-1.92]	0.8	1.3 [0.72-2.35]	0.4

Study participants were stratified into 3 equal groups according to low, medium or high levels of antigen-specific antibodies. Hazard ratios were calculated comparing those with low versus medium levels of antibodies or low versus high levels of antibodies for the risk of clinical malaria over 6 months of follow-up; analysis was based on first episode only. Unadjusted hazard ratios (HR), and adjusted (age-adjusted and location-adjusted) hazard ratios hazard ratios (aHR) were calculated and interquartile ranges [IQR] are shown. Statistical significance is indicated as p.

Supplementary figure 1

A

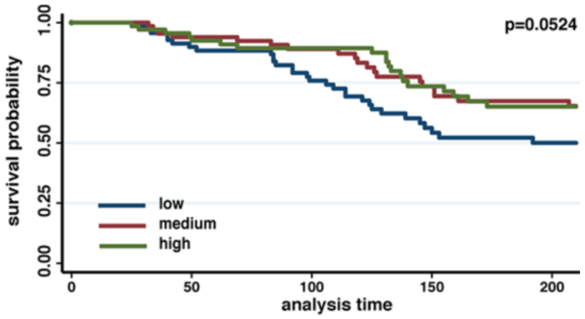

B

	uHR (95%CI)	p	aHR (95%CI)	p
LvM	0.573 [0.32-1.02]	0.058	0.568 [0.32-1.01]	0.055
LvH	0.569 [0.32-1.01]	0.055	0.602 [0.33-1.09]	0.094

Risk of malaria based on in vitro growth inhibition of P. falciparum as measured using growth inhibition assays (GIA)

A. Children were stratified into groups of high (green), medium (red) and low (blue) responders according to growth inhibitory activity as measured by standard GIA (complement-independent), where green is high inhibition (i.e. low growth), red is medium inhibition (i.e. medium growth), and blue is low inhibition (i.e. high growth). Kaplan-Meier survival curves show risk of clinical malaria. Observation time was 210 days. Statistical significance was determined by Wilcoxon test for differences between all three groups.

B. Hazard ratios were calculated using Cox proportional harzards model based on first episode only. Unadjusted hazard ratios (HR), and adjusted (aHR; age-adjusted and location-adjusted) hazard ratios hazard ratios were calculated.

Supplementary figure 2:

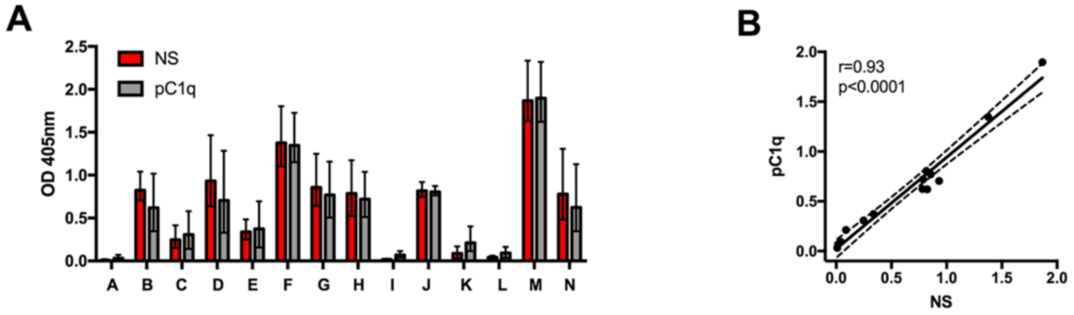

Correlation between the fixation of C1q when using purified C1q or normal serum as the source of complement.
The antibody-dependent fixation of C1q was compared in ELISA based assays using purified C1q (pC1q) or normal serum (NS) as the source of complement on merozoite antigen MSP2. (**A**) Bars show the mean of three assays, each performed in duplicate, error bars represent the range. A-N on the x-axis indicate serum samples used as a source of antibodies from donors A-N. C1q fixation was measured as OD at 405nm. (**B**). Correlation between results using purified C1q (y-axis) versus normal serum, NS (x-axis) as the source of complement using results from A. r= Spearman's rho. Dotted lines show 95% confidence intervals of the fitted line (generated by non-linear regression).

Supplementary figure 3:

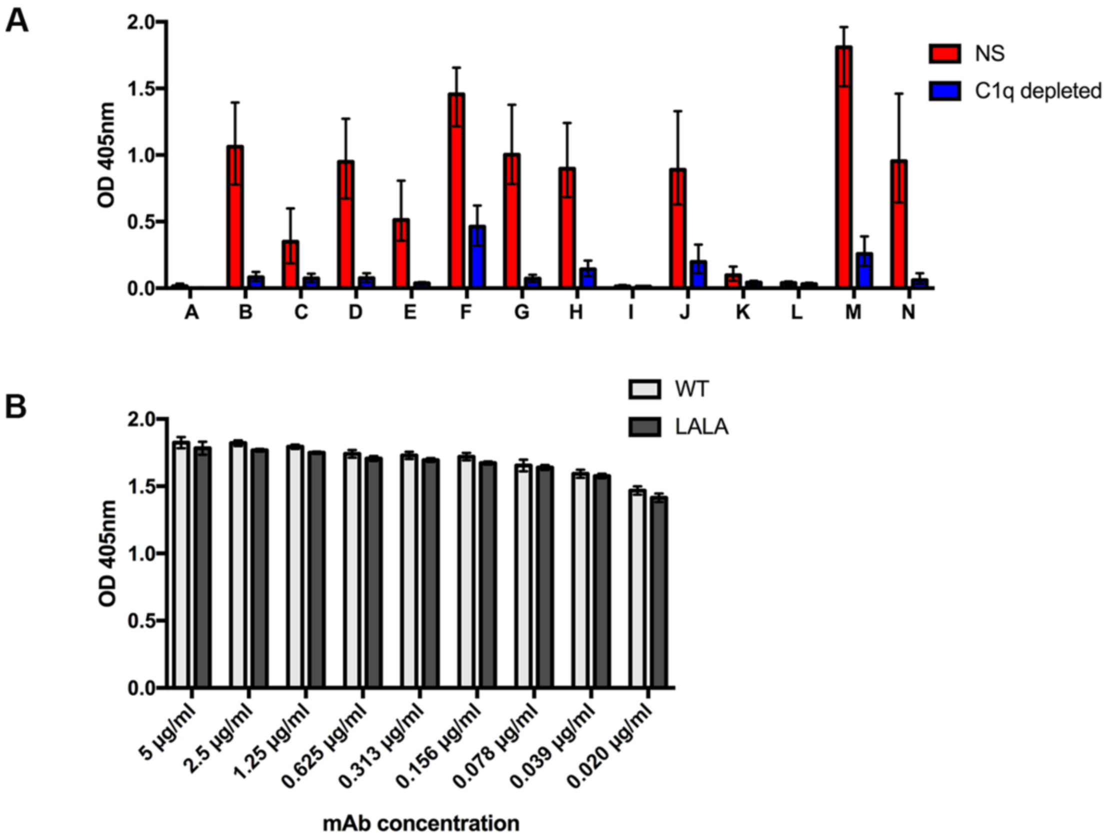

(**A**) Antibody-dependent C1q fixation was determined using heat inactivated serum (HIS), normal serum (NS) or C1q depleted serum as the source of C1q in ELISA based assays and MSP2 (FC27) as the target antigen. Results are shown as mean ODs from 3 independent assays, each run in duplicate. Error bars represent the range of the results. x-axis: serum samples A-N used as source of antibodies. C1q fixation is strongly reduced with the use of HIS and C1q-depleted serum. (**B**) WT and LALA mutant monoclonal antibodies were compared using ELISA for reactivity to recombinant MSP2 (FC27). Results are shown as OD at 405nm. Concentrations of antibody are indicated on the x-axis as [µg/ml]. Shown are the means of two duplicates and the error bars show the range.

Supplementary figure 4:

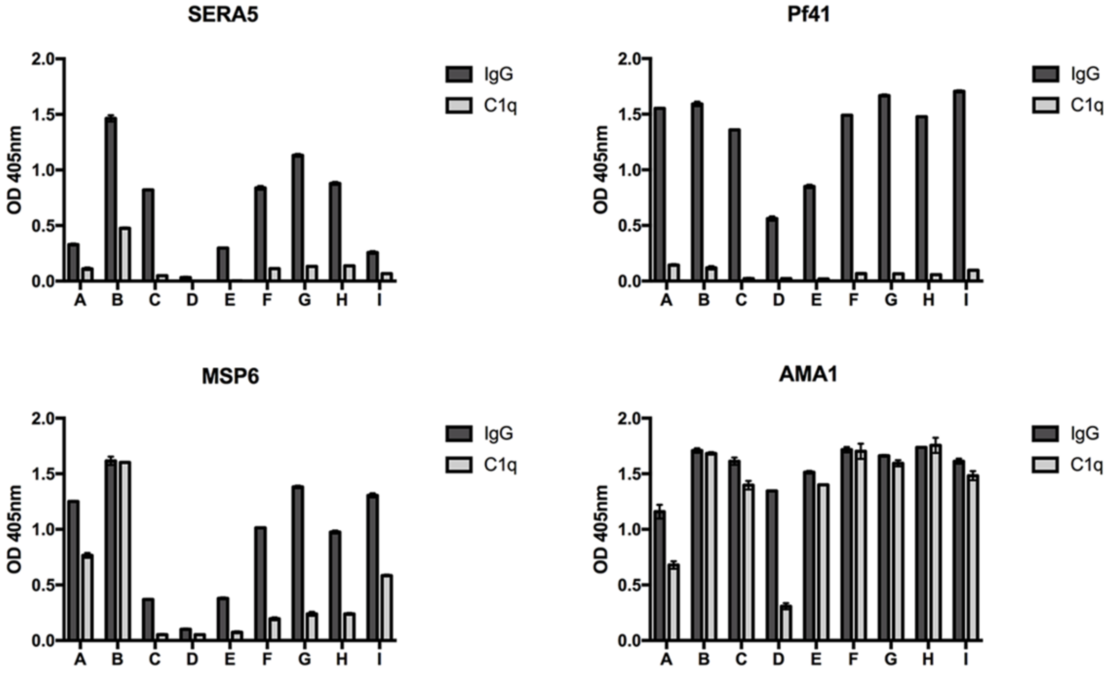

Screen of antigens for presence of total IgG and C1q fixation

In order to select a panel of antigens to be investigated in the longitudinal cohort for the presence of C1q-fixing antibodies, antigens were tested with selected samples from a cross-sectional study including PNG adults. Antigens that showed the presence of significant C1q-fixing antibodies (E.g. MSP6, AMA1) were tested in the longitudinal Mugil cohort. Some antigens had very little reactivity in complement fixing assays and were not evaluated in the longitudinal cohort study (E.g. P41, SERA5).

Supplementary figure 5:

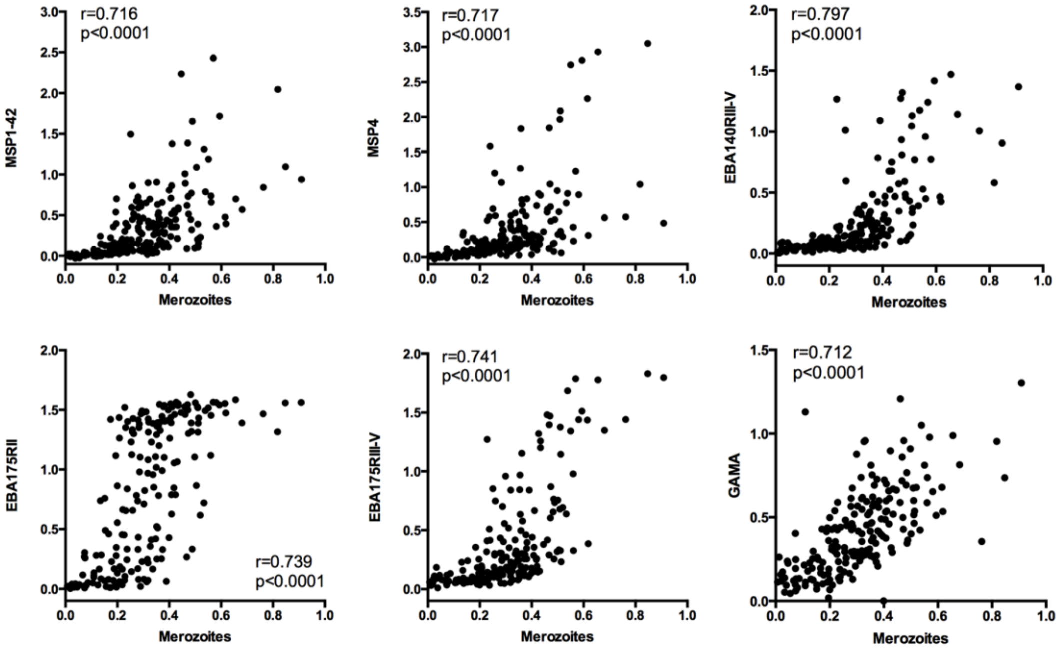

Examples of correlations between complement fixation on whole merozoites and individual merozoite antigens

The fixation of C1q on whole merozoites was compared to the fixation of C1q on individual merozoite antigens. As representative results, comparisons between whole merozoites (on the x-axes) and MSP1-42, MSP4, EBA140RIII-V, EBA175RII, EBA175RIII-V and GAMA (on the y-axes) are shown. Correlations were calculated using Spearman's rho. Statistical significance is indicated as p-value for each comparison.

Supplementary figure 6:

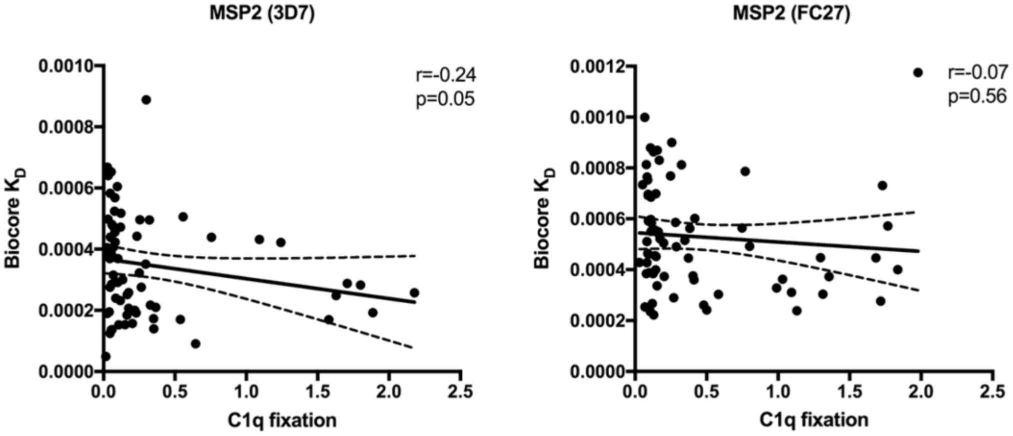

Affinity of MSP2-specific antibodies does not correlate with complement fixation
C1q fixation (y-axis) on the 3D7 or FC27 allele of MSP2 was plotted against the affinity of antibodies against the respective MSP2 allele. Affinity was measured using surface plasmon resonance (Biacore), and is expressed as K_d Samples used were from PNG adults and children (cross-sectional cohort, n=70). Correlations were assessed using Spearman's rho (r). P values indicate statistical significance and are two-tailed. Linear regression was used to generate a fitted curve. Dashed lines represent 95% confidence intervals.

Supplementary figure 7:

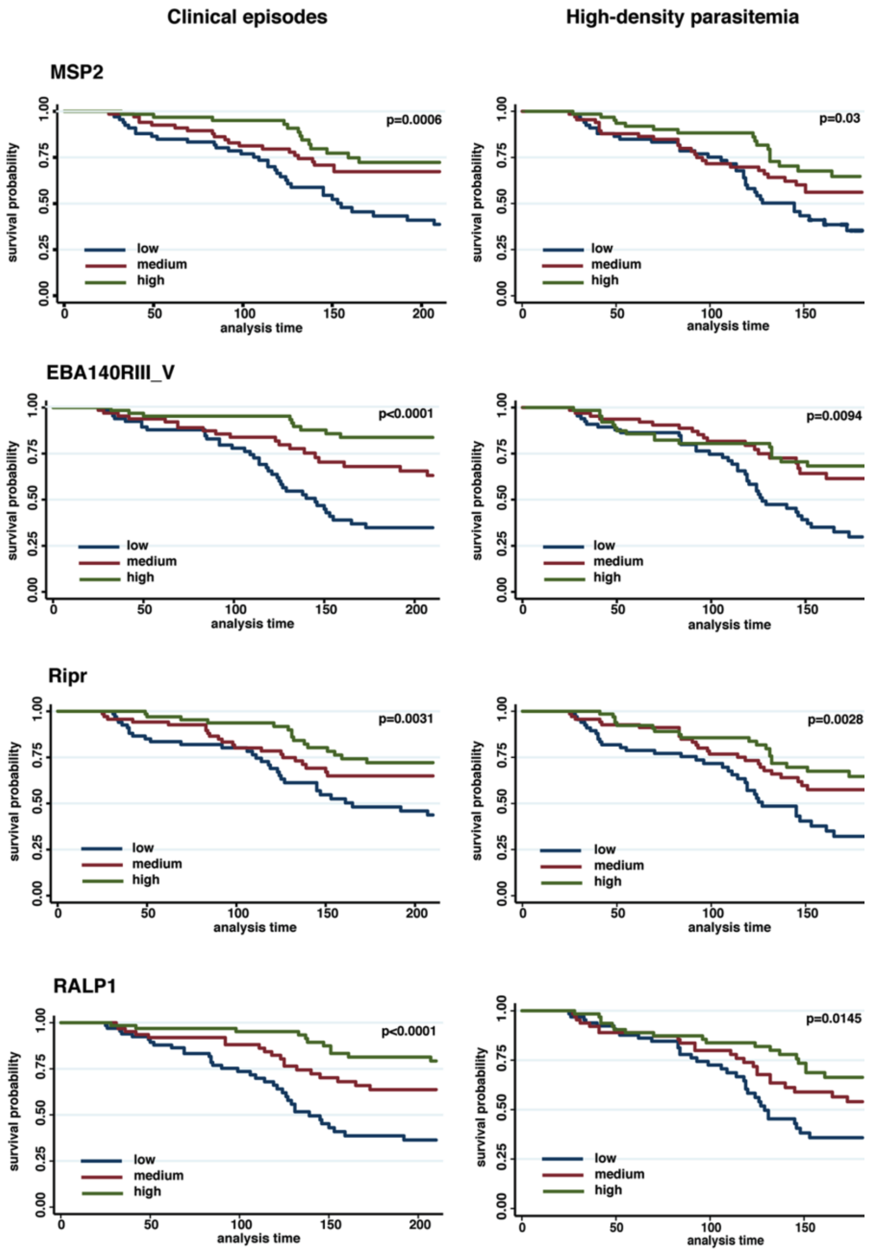

High levels of complement fixing antibodies are associated with protection over time; examples of Kaplan-Meier curves for individual antigens

Study participants were stratified into groups of high (green), medium (red) and low (blue) responders according to antibody dependent C1q fixation. Kaplan-Meier survival curves show proportion of subjects remaining malaria-free over time, for either symptomatic malaria or high-density parasitemia. Observation time was 210 days for time to symptomatic malaria, and 181 days for time to high-density parasitemia. Statistical significance was determined by Wilcoxon test for differences between all three groups.

Supplementary figure 8

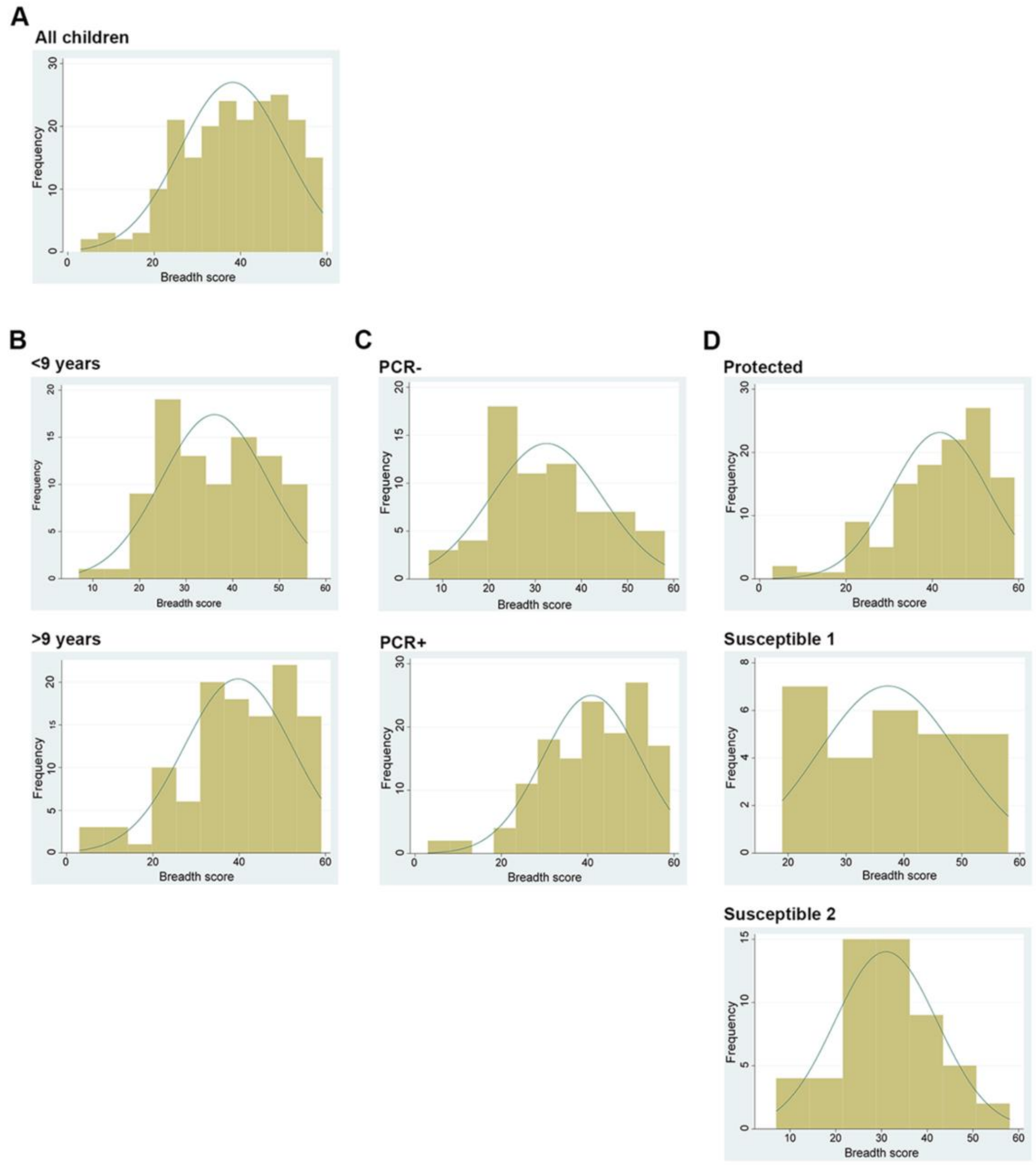

Breadth of antibodies to multiple antigens and clinical associations

The breadth score is a measure of the breadth of complement-fixing antibodies against merozoite antigens. For each antigen tested, responses were stratified into tertiles according to low, medium and high antibody levels, and assigned a score of 1, 2 and 3, respectively. Each childs' scores were added up to result in the breadth score. The histograms are showing the breadth score distribution of (A) all children, (B) all children stratified by age (< or > 9years of age), (C) parasitemic status at enrolment (PCR- or PCR+), or (D) by number of malaria episode during follow up, where protected children experience no malaria episode, children classified as susceptible 1 experienced one malaria episode, and children classified as susceptible 2 experienced 2 or more malaria episodes. The median breadth score was higher in older children and children who were PCR-positive at enrolment. Children classified as protected

had a higher median breadth score compared to children classified as susceptible 1 and 2.

1. Boyle MJ, Reiling L, Feng G *et al.* Human antibodies fix complement to inhibit Plasmodium falciparum invasion of erythrocytes and are associated with protection against malaria. *Immunity* 42(3), 580-590 (2015).
