# Supplementary material for: Targets of complement-fixing antibodies in protective immunity against malaria in children
Source: Nat Commun. 2019 Feb 5;10:610. doi: 10.1038/s41467-019-08528-z (PMC6363798; doi:10.1038/s41467-019-08528-z)
Supplement: Supplementary file 4 — Reporting Summary [file 41467_2019_8528_MOESM4_ESM.pdf]

nature research

NCOMMS-18-28857
Reiling L., et al.
Corresponding author(s): Corresponding Author - James Beeson

Reporting Summary

Nature Research wishes to improve the reproducibility of the work that we publish. This form provides structure for consistency and transparency in reporting. For further information on Nature Research policies, see Authors & Referees and the Editorial Policy Checklist.

Statistical parameters

When statistical analyses are reported, confirm that the following items are present in the relevant location (e.g. figure legend, table legend, main text, or Methods section).

n/a	Confirmed	
☐	☒	The exact sample size (*n*) for each experimental group/condition, given as a discrete number and unit of measurement
☐	☒	An indication of whether measurements were taken from distinct samples or whether the same sample was measured repeatedly
☐	☒	The statistical test(s) used AND whether they are one- or two-sided<br>*Only common tests should be described solely by name; describe more complex techniques in the Methods section.*
☐	☒	A description of all covariates tested
☐	☒	A description of any assumptions or corrections, such as tests of normality and adjustment for multiple comparisons
☐	☒	A full description of the statistics including central tendency (e.g. means) or other basic estimates (e.g. regression coefficient) AND variation (e.g. standard deviation) or associated estimates of uncertainty (e.g. confidence intervals)
☐	☒	For null hypothesis testing, the test statistic (e.g. *F*, *t*, *r*) with confidence intervals, effect sizes, degrees of freedom and *P* value noted<br>*Give P values as exact values whenever suitable.*
☒	☐	For Bayesian analysis, information on the choice of priors and Markov chain Monte Carlo settings
☒	☐	For hierarchical and complex designs, identification of the appropriate level for tests and full reporting of outcomes
☐	☒	Estimates of effect sizes (e.g. Cohen's *d*, Pearson's *r*), indicating how they were calculated
☐	☒	Clearly defined error bars<br>*State explicitly what error bars represent (e.g. SD, SE, CI)*

Our web collection on statistics for biologists may be useful.

Software and code

Policy information about availability of computer code

Data collection	*Provide a description of all commercial, open source and custom code used to collect the data in this study, specifying the version used OR state that no software was used.*
Data analysis	Demographic, clinical, and immunologic data was managed and analysed in STATA statistical packaged, Prism Graphpad, and R open source software, as described in the manuscript

For manuscripts utilizing custom algorithms or software that are central to the research but not yet described in published literature, software must be made available to editors/reviewers upon request. We strongly encourage code deposition in a community repository (e.g. GitHub). See the Nature Research guidelines for submitting code & software for further information.

Data

Policy information about availability of data

All manuscripts must include a data availability statement. This statement should provide the following information, where applicable:

- Accession codes, unique identifiers, or web links for publicly available datasets
- A list of figures that have associated raw data
- A description of any restrictions on data availability

All data are summarised in relevant tables and figures, and supplementary tables and figures, as described in the manuscript. Data used for plotting figures are

provided as a Supplementary Information file. Access to data from the longitudinal cohort used for analysis of associations with clinical parameters is available from the authors for research purposes only by request and will be subject to conditions set by the relevant human research ethics committee and regulatory committees

Field-specific reporting

Please select the best fit for your research. If you are not sure, read the appropriate sections before making your selection.

☒ Life sciences ☐ Behavioural & social sciences ☐ Ecological, evolutionary & environmental sciences

For a reference copy of the document with all sections, see nature.com/authors/policies/ReportingSummary-flat.pdf

Life sciences study design

All studies must disclose on these points even when the disclosure is negative.

Sample size: The sample size was pre-determined by the size of the cohort study, which was n=206 participants. We had already established that there was a significant association between complement-fixing antibodies to intact merozoites and malaria risk during follow-up; this provided the rationale that there was sufficient power to assess protective associations for antibodies to specific antigens and combinations of antigens

Data exclusions: No data were actively or selectively excluded. In some analyses, there were missing data for a small number of participants, and this is indicated in the manuscript as appropriate

Replication: All samples were tested in assays in duplicate, as described in the manuscript. Some assays were performed twice in duplicate, as indicated

Randomization: This study included an observational cohort study, and there was no randomization. In participating villages, all children of the target age range (with discussion and consent from their parents/guardians) were invited to participate in the study.

Blinding: This was an observational study; therefore there were no subject allocations or formal blinding. However, all samples were coded and tested blind to the clinical data.

Reporting for specific materials, systems and methods

Materials & experimental systems

n/a	Involved in the study	
☐	☒	Unique biological materials
☐	☒	Antibodies
☒	☐	Eukaryotic cell lines
☒	☐	Palaeontology
☒	☐	Animals and other organisms
☐	☒	Human research participants

Methods

n/a	Involved in the study	
☒	☐	ChIP-seq
☒	☐	Flow cytometry
☒	☐	MRI-based neuroimaging

Unique biological materials

Policy information about availability of materials

Obtaining unique materials: Includes 1) plasma samples collected from children exposed to malaria - these are not available for distribution because of requirements of ethics and regulatory committees. 2) Antibodies - rabbit antibodies raised against complement factor C1q. Small aliquots are available for research purposes on reasonable request. 3) Recombinant proteins of P. falciparum. These may be available to others but would need to be produced for specific purposes. 4) Merozoites isolated from in vitro cultures of P. falciparum. Detailed protocols are publicly available for others to use, and P. falciparum cell lines used for merozoite isolation are freely available.

Antibodies

Antibodies used: Antibodies used are described in the manuscript. Specific product details for antibodies obtained from commercial suppliers are provided in the manuscript

Validation: Rabbit antibodies raised against complement factor C1q, and commercial antibodies to complement factors were validated using western blotting and ELISA. Antibodies to IgG, IgM, and IgG subclasses (commercial suppliers) were validated using purified

antibodies in ELISA. Recombinant proteins were expressed and purified using established methods and previously validated. Protein integrity and purity checked by SDS PAGE gels. Merozoites were isolated from in vitro culture and confirmed by flow cytometry.

Human research participants

Policy information about studies involving human research participants

Population characteristics	All details are provided in the manuscript. The study populations were previously described in extensive detailed in a prior epidemiologic study which is indicated in the manuscript.
Recruitment	All children in study villages were able to participate in the study. There were no specific selection criteria other than age, and males and females were equally represented, as described in the manuscript
